# Study on Characteristics of Chemokine CXCL10 Gene Cloned from cDNA Expression Library of Ujumqin Sheep

**DOI:** 10.1155/2013/217942

**Published:** 2013-09-28

**Authors:** P. F. Hu, X. C. Li, N. Lei, X. Y. Lan, Q. J. Zhao, W. J. Guan, Y. H. Ma

**Affiliations:** ^1^Institute of Animal Sciences, Chinese Academy of Agricultural Sciences, Beijing 100193, China; ^2^College of Animal Science and Technology, Northwest A & F University, Shaanxi Key Laboratory of Molecular Biology for Agriculture, Yangling, Shaanxi 712100, China

## Abstract

Chemokines were a major regulator of body's inflammatory and immune responses. In this study, the cDNA fragment of chemokine CXC ligand 10 (CXCL10) was cloned from the Ujumqin sheep ear marginal tissue cDNA expression library; the CXCL10 gene had 103 amino acids and a molecular weight of 11.47 kDa, and it shared a high homology among cattle, sheep, and goat, while a low homology compared with mouse. The CXCL10 protein had 4 conservative cysteine residues, located in 28, 30, 55, and 72 sites. The expression pattern and intracellular distribution of recombinant CXCL10 proteins in Ujumqin sheep fibroblast cells showed that there were green fluorescence signals both in cytoplasm and nucleolus after 24 h of transfection, the number of positive cells was increased with time, the peak level of fluorescence signal was reached after 48 h of transfection and the transfection efficiency was 33.3%; there was a significant decrease in fluorescence intensity after 72 h of transfection. Expression of recombinant CXCL10 gene in *Escherichia coli* had a time- and temperature-dependency on the amount of protein expression, and a small quantity of inducer was needed.

## 1. Introduction

Ujumqin sheep, a larger version of the Mongolian, was found in Inner Mongolia, China. These sheep have a type of coarse wool commonly called “carpet wool” and have an ability to deposit fat in the tail; thus, they could adapt to the unfavourable local environmental conditions of the north and northwest pastoral grasslands. In the year of 2000, they were listed as one of the 78 nationally protected domestic animals by the Chinese government. 

In the past years, tremendous success was achieved with the molecular genetics study of sheep [1–3]. Recently, functional gene study became one of the hottest issues [4, 5]. Our group have been engaged in the study of molecular mechanism on the growth and development of sheep for many years; a series of genes were found, such as NADH dehydrogenase subunit 2(ND2), subunit 4 (ND4) [6], TSK21 [7], cytochrome c oxidase subunit VIa polypeptide 1-like gene (NCBI Accession no. GU585577); now we are paying more attention to chemokines of Ujumqin sheep because of their multiple biological functions especially in the mechanism of development, prevention and treatment of disease.

Chemokines were a superfamily of proteins with low molecular weight (8–15 kD), composed of 70–90 amino acids with homology of 20–70% among different species, and had 4 conservative cysteine residues. Chemokines could selectively attract and activate leukocytes and a variety of other types of cells. They still played a role in the process of infection, directional migration, and differentiation of immune cells. Chemokines were a major regulator of body's inflammatory and immune responses; they were also involved in dendritic cell maturation, macrophage activation, and neutrophil degranulation [8]. According to the number and arrangement of N-terminal conserved cysteine, chemokines could be divided into CXC, CC, C, and CX3C families [9]; they could also be divided into ELR and non-ELR types based on the existence of ELR (glutamic acid, Leucine, and Arginine) in the protein constitution.

Chemokine CXC ligand 10 (CXCL10) was a member to CXC family, also known as interferon-inducible protein 10 (IP-10), CXCL10 was a 10 kDa protein which could selectively attract and activate lymphocytes, and CXCL10 binded to the receptor CXCR3 and regulated immune responses through the activation and recruitment of leukocytes, such as T cells, eosinophils, and monocytes [10]. Obviously, the further study on the CXCL10 gene had important significance in understanding the mechanism of development, gene function, and prevention and treatment of disease, that was also the main purpose of this study. 

The cDNA library construction and analysis were considered to be an indispensable tool for functional genomic analysis as it provided much more detailed information on the genomic mechanisms underlying diverse processes of the organism [11]. The SMART technique provided a method for producing high-quality and full-length cDNA libraries that preserved the complete 5′ terminal sequence of mRNA [12]. Construction of Ujumqin sheep cDNA library for the protection of genetic resources as well as study of gene function had an important genetic significance. Construction of cDNA expression library could not only protect endangered treasure of biological resources, but also provide molecular markers linkage map of the building used by probes; more importantly, cDNA expression library could be used to separate full-length genes and then to carry out gene function study. At present, the sequence information of CXCL10 gene for human [13], mouse [14], cattle [15], sheep [16], goat [17], and pig [18] had been reported. However, CXCL10 gene from the Ujumqin sheep had not been reported, especially in aspect of *in vitro* expression characteristics. 

Full-length cDNA sequence of CXCL10 was usually obtained using the rapid amplification of cDNA ends (RACE) method [19, 20]. In this study, the cDNA of CXCL10 gene was amplified from the Ujumqin sheep ear marginal tissue cDNA expression library; then the sequence characteristics, structure, and homology of the protein encoded by the cDNA were analyzed; it was carried out to evaluate *in vitro *expression characteristics of CXCL10 gene from Ujumqin sheep. This study could provide scientific data and means for postulating the immune mechanism, disease resistance research, and the development of functional genes from the Ujumqin sheep. 

## 2. Materials and Methods

### 2.1. Animals and cDNA Expression Library Construction

All procedures involving animals were approved by the animal care and use committee at the institution where the experiment was conducted. All procedures involving animals were approved and authorized by the Chinese Ministry of Agriculture.

Ear margin tissue samples were collected from Ujumqin sheep at the Conservation Center of Institute of Animal Sciences, Chinese Academy of Agricultural Sciences (Beijing, China). The samples were frozen in liquid nitrogen and then used for RNA isolation. cDNA expression library was constructed using SMART technology (SMART cDNA Library Construction Kit, Clontech); titer of the unamplified library, percentage of recombinant clone, and titer of the amplified library were determined subsequently.

### 2.2. Cloning of CXCL10 Gene by Screening of the cDNA Expression Library

CXCL10 gene was obtained by screening of the cDNA expression library using PCR method. The PCR primers were designed by Primer Premier 5.0, based on the mRNA sequence of CXCL10 gene from Ovis aries (NM 001009191.1); the specific primers of cDNA sequence were as follows:

 P1: 5′-TGCAGCACCATGAACAAAAT-3′,
 P2: 5′-GTGATTATGCCTCTTTCTGTGTTC-3′.

The PCR products were cloned into TA-cloning vector pGEM-T Easy (Promega). Plasmid DNA was sequenced by Huada Zhongsheng Scientific Corporation (Beijing, China).

### 2.3. Overexpression of Recombinant CXCL10 Gene in Fibroblast Cells

The DNA fragment of CXCL10 gene was flanked with *Xho I* and *BamH I *sites; after double digestion, the final fragment was inserted into pEGFP-N3 vector (Clontech) for transfection.

Ujumqin sheep fibroblast line which has been established in our laboratory was used as target cells [21]. Cells were seeded in 24-well plates and transfected with the plasmid DNA of CXCL10 gene (pEGFP-N3-CXCL10) by Lipofectamine 2000 (Invitrogen). The medium was refreshed 6 h after transfection, and cells were observed 24, 48, and 72 h after transfection using Nikon TE-2000-E inverted confocal microscope with excitation wavelengths of 488 nm to determine the transfection efficiency and morphology of positive cells. For each experimental group, images were captured from 10 visual fields to determine the total and positive cell counts in each field for the calculation of transfection efficiencies.

### 2.4. Expression Characteristics of Recombinant CXCL10 Gene in *Escherichia coli*

The DNA fragment of CXCL10 gene was flanked with *BamH I* and *Xho I* sites; after double digestion, the final fragment was inserted into pGEX-4T-1 vector (GE Healthcare) for protein expression.

The constructed expression plasmid was transformed into BL21 (DE3) competent cells (Tiangen), and the recombinant protein production was carried out using autoinduction method. Briefly, proteins were expressed in cells by induction with isopropyl-1-thio-*β*-D-galactopyranoside (IPTG). Integrality of recombinant proteins was tested by Western blot, the expression condition was optimized, and inducer concentration, induction time, and temperature were tested respectively, for high-level protein expression [22]. The results were confirmed using SDS-PAGE.

### 2.5. Data Analysis

Homology study of the Ujumqin sheep CXCL10 gene compared with the gene sequences of other species was performed using Blast 2.1 (http://www.ncbi.nlm.nih.gov/blast/). ORF of the DNA sequence was searched using ORF finder software (http://www.ncbi.nlm.nih.gov/gorf/gorf.html). The values of WM and pI were computed using the Compute pI/Mw tool (http://www.expasy.org/tools/pi_tool.html). Protein structure of the CXCL10 gene sequence cloned was analyzed using PredictProtein software (http://emboss.bioinformatics.nl/cgi-bin/emboss). Multiple sequence alignment was performed by software DNAstar Lasergene and DNAMAN 6.0. 

## 3. Results and Analysis

### 3.1. Amplification and Detection of the Constructed cDNA Expression Library

**Figure 1:**
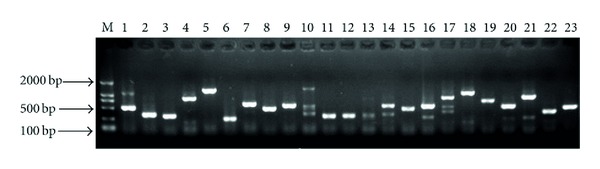
PCR products of the random clones. M: 2000 bp, 1–23: cDNA fragment.

Titers of the unamplified and amplified library were determined by counting the number of colonies according to the protocol of cDNA Library Construction Kit; the results showed that unamplified and amplified library had a titer of 8.24 × 10^6^ pfu/mL and 1.60 × 10^10^ pfu/mL, respectively. To test for ligation efficiency in the amplified library, the percentage of recombinant clones was determined by screening 96 cDNA inserts using PCR method; the results showed that the ligation of the cDNA to the *λ*TriplEx2 Vector was 93.75% recombinants, and the average length of cDNA inserts was 1.0 kb (Figure 1). The full-length cDNA library constructed from Ujumqin sheep conformed to the requirements of a standard library [23].

### 3.2. Analysis of the cDNA of CXCL10 Gene from Ujumqin Sheep

**Figure 2:**
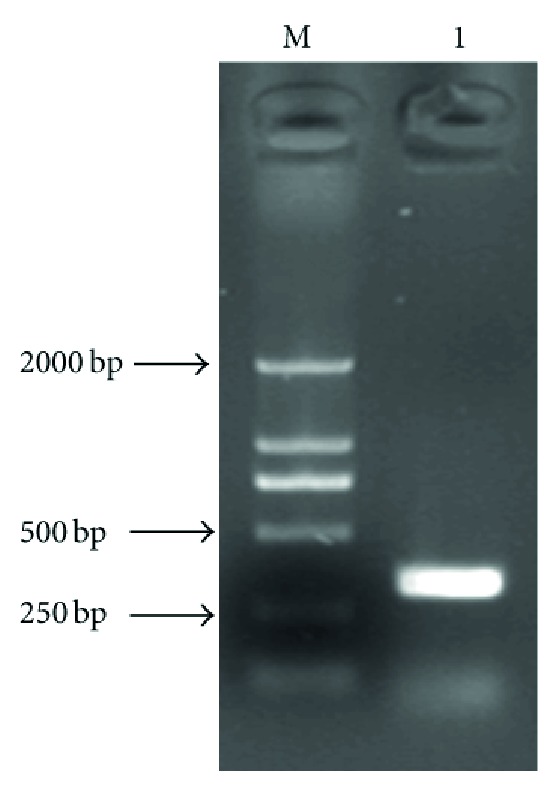
PCR products of the Ujumqin sheep CXCL10 gene. M: molecular marker DL2000, 1: amplified CXCL10 gene.

The cDNA fragment with 309 bp in size was amplified from the cDNA expression library (Figure 2). On the basis of the high identity, it was concluded that the cDNA isolated was the cDNA encoding the Ujumqin sheep CXCL10 gene. The CXCL10 gene sequence was submitted to Genbank (Accession no. HM017954). 

As determined by homology analysis, the nucleotide sequence of CXCL10 gene cloned from the Ujumqin sheep shared a homology with those of human, mouse, cattle, sheep, goat, and pig of 77.46%, 70.48%, 96.76%, 100%, 98.71%, and 87.94%, respectively (see Figure S1a in Supplementary material available online at http://dx.doi.org/10.1155/2013/217942); the homologies for amino acid sequences were 84.62%, 80.77%, 99.02%, 100%, 99.02%, and 93.27%, respectively (Figure S1b). It was indicated that CXCL10 gene shared a high homology among cattle, sheep, and goat; there was also a high homology of sheep compared with human and pig, while homology between sheep and mouse was the lowest. 

### 3.3. Prediction and Analysis of Primary Structure, Protein Functional Sites, and Advanced Structure in CXCL10 of the Ujumqin Sheep

Primary structure analysis revealed that the molecular weight of the putative CXCL10 protein of the Ujumqin sheep was 11.47 kDa with a theoretical pI 10.20. Like most chemokines, CXCL10 protein of the Ujumqin sheep had 4 conservative cysteine residues, located in 28, 30, 55, and 72 sites, in which the first 2 cysteine residues were separated by threonine (Figure S2); it was demonstrated that the sequence characteristics of CXCL10 gene which we cloned from Ujumqin sheep were accordant with the members to CXC family. While the highest content is the Leu residues (11.77%), far higher than other amino acids and without Asp, His, Trp, and Tyr residues (Figure S3), it was indicated that Ujumqin sheep CXCL10 protein was ELR type based on the existence of ELR (glutamic acid, Leucine, and Arginine) in the protein constitution. The secondary structure analysis of CXCL10 protein indicated that the protein was 39.5% in helix, 18.6% in sheet, 39.5% in turns, and 20.9% in coil. 

The water unsoluble property of CXCL10 protein limits its isolation, purification, and crystal growth, so it was difficult to determine the structure of CXCL10 protein. Transmembrane helices prediction was an important application in bioinformatics. In this study, tmap in the EMBOSS database and TMHMM 2.0 were comprehensively used in the prediction of transmembrane domain of CXCL10 protein. The result showed that high hydrophobicity occurred in the first 28 residues, indicating a probable transmembrane domain (Figure S4a).

Signal peptide located in the N-terminus of secretory protein, and there was approximately 20 residues signal peptides in a typical chemokine; in this study, signal peptide position and cleavage site of CXCL10 protein of Ujumqin sheep were predicted by SignalP 2.0, the signal peptide of CXCL10 protein was consisted of a positive charge region, a hydrophobic region, and a polar region (Figure S4b), prediction of cleavage site was evaluated by Y-score maximum, the result showed that cleavage site of CXCL10 protein was between pos. 19 and 20, and mean S-score in pos. 1–19 was 0.928; it could be indicated that CXCL10 protein was secretory protein, and there was a signal peptide sequence in the N-terminal of CXCL10 protein. 

In most cases, two pairs of double sulfur bands consisted by 4 conservative cysteine residues formed special structure of chemokine; in this study, spatial structure of CXCL10 protein of Ujumqin sheep was predicted by CPHmodels 2.0, and human [24] and mouse [25] CXCL10 protein derived from PDB protein database were taken as template; after Magic Fit by Swiss-PdbViewer4.0, the result showed that the spatial structure of CXCL10 protein was accordant with secondary structure predicted by garnier. Ujumqin sheep CXCL10 protein had similar structure with human, while different from mouse (Figure S5).

Topology prediction showed that there was a small cytokines C-x-C subfamily signature in the CXCL10 protein of Ujumqin sheep; these small cytokines were also called intercrines or chemokines. They were cationic proteins of 70–100 amino acid residues that shared four conserved cysteine residues involved in two disulfide bonds. There was also two N-glycosylation sites, one N-myristoylation site, two Casein kinase II phosphorylation sites, and five protein kinase C phosphorylation sites in the CXCL10 protein of Ujumqin sheep (Figure S6). 

### 3.4. Overexpression of Recombinant CXCL10 Gene in Fibroblast Cells

The expression pattern and intracellular distribution of recombinant CXCL10 protein in Ujumqin sheep fibroblast cells were analyzed after transfection with the plasmid pEGFP-N3-CXCL10. pEGFP-N3 encodes a red-shifted variant of wild-type GFP which has been optimized for brighter fluorescence and higher expression in somatic cells of livestock and poultry, which had been demonstrated in our early studies [26, 27]. CXCL10 gene cloned into the MCS could be expressed as fusions to the N terminus of EGFP. 

**Figure 3:**
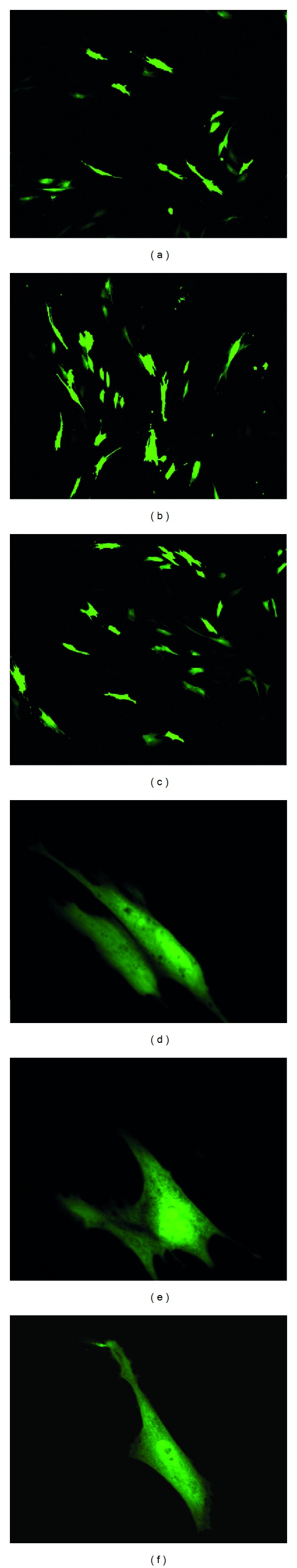
Transfection of recombinant pEGFP-N3-CXCL10 in Ujumqin sheep fibroblast cells. (a), (b), (c) Transfection after 24 h, 48 h, and 72 h, respectively (100x). (d), (e), (f) Transfection after 24 h, 48 h, and 72 h, respectively (400x).

In the initial stage of transfection, the majority of the cells had no significant change in morphology; some of the cells were shrinking and the number of live cells was decreased to some extent, there were green fluorescence signals both in the cytoplasm and nucleolus of transfected cells after 24 h (Figure 3(a)), the number of positive cells was increased with time, and they reached the peak level after 48 h of transfection (Figure 3(b)). The transfection efficiency was 33.3%; there was a significant decrease in fluorescence intensity after 72 h of transfection (Figure 3(c)). Fluorescence intensity was stronger in nucleolus than in cytoplasm during the transfection (Figures 3(d), 3(e), and 3(f)). The high level of recombinant CXCL10 gene expression in fibroblast cells could last for 1 week, indicating that gene duplication, transcription, protein synthesis, and modification were highly effective in transfected cells, and there was no significant effects of recombinant proteins on cell growth and proliferation.

### 3.5. Expression of Recombinant CXCL10 Gene in *Escherichia coli*

There was target protein expression after 6 h induction by 1 mmol/L IPTG and molecular weight of target protein was 37.47 kD. GST occurred naturally as a 26 kDa protein that could be expressed in *E. coli* with full enzymatic activity, while CXCL10 protein had a molecular weight of 11.47 kDa. Fusion proteins that possess the complete amino acid sequence of GST also demonstrated GST enzymatic activity and could undergo dimerization similar to that observed in nature; it was concluded that the recombinant protein was CXCL10 protein after Western blot analysis (data were not shown). 

Yield of fusion protein was highly variable and was affected by the nature of the fusion protein, the host cell, and the culture conditions used during the optimization of expression conditions. In this study, samples from evaluations of media, growth temperature, culture density, induction conditions, and other variables were processed successively. 

**Figure 4:**
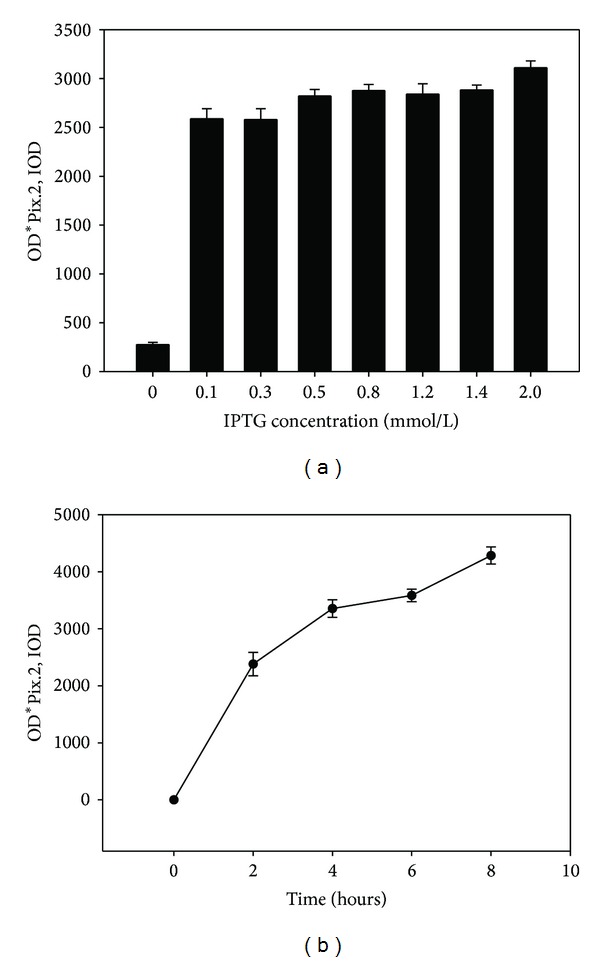
Effect of IPTG concentration and induction time on recombinant protein expression. (a) Protein expression with different IPTG concentrations, (b) protein expression with different induction time.

Protein expression was increased with temperature rising: the highest amount of protein expression appeared at the temperature of 37 degrees; then the yield of recombinant protein decreased as the temperature raised to 40 degrees (data were not shown). When the IPTG concentration was 0.1–2.0 mmol/L, there was no significant change in recombinant CXCL10 protein expression under 37-degree induction (Figure 4(a)). It was concluded that recombinant proteins were completely expressed by induction with IPTG. Without inducer, there was little recombinant protein expression. When the inducer concentration was 0.1, 0.3, 0.5, 0.8, 1.2, 1.4, and 2.0 mM, respectively, the expression of the recombinant proteins was initiated, but there were no significant changes in expression, indicating that a small quantity of inducer was needed when the protein expression was in control of the tac promoter. When the induction of recombinant CXCL10 protein was performed with the IPTG at 0.1 mM for 0, 2, 4, 6, and 8 h at 37 degrees, respectively, protein expression was increased with induction time (Figure 4(b)).

In summary, the optimal inducer concentration for the expression of recombinant CXCL10 protein was 0.1 mM, the optimum induction time was 8 h, and the highest amount of protein expression was achieved at 37 degrees. 

## 4. Discussion

### 4.1. Cloning of CXCL10 Gene from SMART cDNA Library of the Ujumqin Sheep

The SMART library provided a useful resource for the functional genomic study of Ujumqin sheep and could present some new molecular material for this species as well. In this study, the results indicated that the length of CXCL10 fragment cloned from cDNA library was 309 bp, encoding 103 amino acids, with a molecular weight of 11.47 kDa. Like most chemokines, CXCL10 protein of the Ujumqin sheep had 4 conservative cysteine residues; CXCL10 gene which we cloned from Ujumqin sheep was accordant with the members to CXC family. 

Bioinformatical analysis of CXCL10 gene was required, which could provide more valuable information in further studies; for example, basic information could be illustrated by DNA chromosome location, introns/exons, and ORF analysis; gene regulation mechanism could be identified by prediction of promoter, CpG island, and transcription factor analysis; characteristics of gene encoding protein could be determined by main properties, subcellular localization, and antigenic sites analysis; gene functions could be further determined by similarity search, function sites, and structure analysis. It was reported that porcine CXCL10 protein has high homology with the CXCL10 of five species—dog (87%), human (84%), monkey (84%), mouse (75%), and rat (70%); the phylogenetic tree analysis revealed that the swine CXCL10 had a closer genetic relationship with the CXCL10 of dog than with those of human, monkey, mouse, and rat [28]. In this study, the nucleotide sequence of CXCL10 gene cloned from the Ujumqin sheep shared a homology with those of human, mouse, cattle, sheep, goat, and pig of 77.46%, 70.48%, 96.76%, 100%, 98.71%, and 87.94%, respectively; the homologies for amino acid sequences were 84.62%, 80.77%, 99.02%, 100%, 99.02%, and 93.27%, respectively. Alignment analysis indicated that CXCL10 gene shared a high homology among cattle, sheep, and goat; there was also a high homology of sheep compared with human and pig, while homology between sheep and mouse was the lowest. 

### 4.2. Overexpression of Recombinant CXCL10 Gene in Ujumqin Sheep Fibroblast Cells

Previous studies had demonstrated that the overexpression of CXCL10 in cancer or tumor cells inhibited cell proliferation of the transfected cells; for example, Nagpal studied the effects of overexpression of CXCL10 in human prostate cancer LNCaP cells. LNCaP cells were transiently transfected with CXCL10 cDNA in pIRES2-EGFP vector, and overexpression of CXCL10 inhibited cell proliferation of the transfected cells by 30%–40% [29]; another study showed that overexpression of CXCL10 inhibited StAR D1 expression, decreased progesterone synthesi, and inhibited cell proliferation of mouse Leydig tumor cell [30], while in our study, it was found that there was no significant effect on the proliferation of Ujumqin sheep fibroblast cells; a possible explanation was that the mechanism of CXCL10 was different in some cell types. 

In the study of Zhao et al. [31], the amplified CXCL10 gene was inserted into the eukaryotic expression vector pcDNA3.1(+), and the recombinant expression vector pcDNA3.1(+)-IP-10 was transfected into NIH 3T3 cells via liposome. The method was accordant with our study, while the cell types were different from our study, and the transfection efficiency was not illustrated. So more details on the *in vitro* expression characteristics of CXCL10 need to be studied. In this study, the CXCL10 gene could be effectively expressed in fibroblast cells. The expression pattern and intracellular distribution of recombinant CXCL10 proteins in Ujumqin sheep fibroblast cells were analyzed after transfection with the plasmid pEGFP-N3-CXCL10. There were green fluorescence signals both in cytoplasm and nucleolus of transfected cells after 24 h, the number of positive cells was increased with time, and they reached the peak level after 48 h of transfection; there was a significant decrease in fluorescence intensity after 72 h of transfection. The transfection efficiency was 22.8%; fluorescence intensity was stronger in nucleolus than in cytoplasm during the transfection. 

### 4.3. Expression of Recombinant CXCL10 Gene in *Escherichia coli*

The lack of immunological reagents for some experimental animals prompted the clone of CXCL10 and the expression of the gene as a recombinant protein. Danesh had cloned and sequenced the genes encoding ferret CXCL10 and then cloned into the pcDNA3.1/His6.V5/TOPO expression vector; recombinant ferret CXCL10 protein was expressed and purified using COS-7 cells [32]. In this study, the DNA fragment of CXCL10 gene was inserted into pGEX-4T-1 vector for protein expression; the pGEX-4T-1 vector was designed for inducible, high-level intracellular expression of genes or gene fragments as fusions with *Schistosoma japonicum* GST [33]. Expression pattern of recombinant CXCL10 gene in *Escherichia coli* was different with induction temperature, inducer concentration, and induction time; the data showed that the recombinant CXCL10 had a time- and temperature-dependency on the amount of protein expression. The data also indicated that a small quantity of inducer was needed when the protein expression was in control of the tac promoter. 

Consequently, analysis of the expression of CXCL10 combined with the knowledge of their functions could facilitate the understanding and allow us to take a glimpse of the overall picture of CXCL10 gene in Ujumqin sheep. The findings could give scientific support and orientation for postulating the immune mechanism, disease resistance research, and the development of functional genes from the Ujumqin sheep. 

## Supplementary Material

Bioinformatical analysis of CXCL10 gene illustrated that CXCL10 gene we cloned from Ujumqin sheep was accordant with the members to CXC family, main properties were determined and gave valuable information for subcellular localization and in vitro expression analysis.
