# Supplementary material for: Study on Characteristics of Chemokine CXCL10 Gene Cloned from cDNA Expression Library of Ujumqin Sheep
Source: Biomed Res Int. 2013 Sep 28;2013:217942. doi: 10.1155/2013/217942 (PMC3804364; doi:10.1155/2013/217942)
Supplement: Supplementary file 1 — Bioinformatical analysis of CXCL10 gene illustrated that CXCL10 gene we cloned from Ujumqin sheep was accordant with the members to CXC family, main properties were determined and gave valuable information for subcellular localization and in vitro expression analysis. [file 217942.f1.pdf]

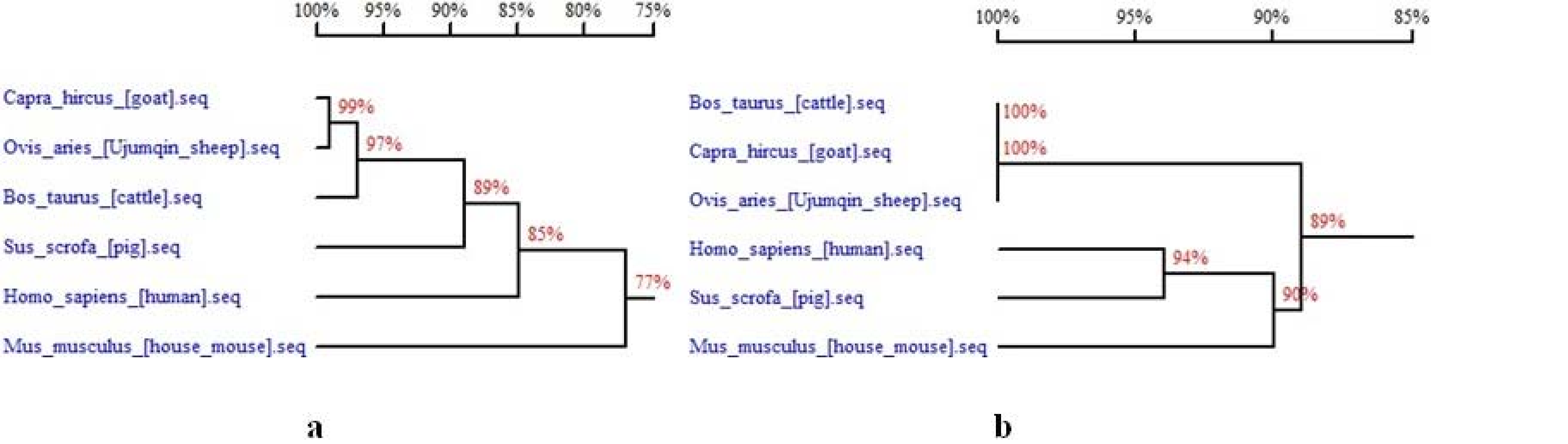

Fig. S1 Homology Tree of chemokine CXCL10 among different species. **a** The homology tree of CXCL10 gene among different species, **b** The homology tree of CXCL10 protein among different species

1	ATG	AAC	AAA	AGT	GGT	TTT	CTT	ATT	TTC	TGC	CTT	ATC	CTT	CTG	ACT	CTG	AGT	CAA	GGC	ATA
1	MET	Asn	Lys	Ser	Gly	Phe	Leu	Ile	Phe	Cys	Leu	Ile	Leu	Leu	Thr	Leu	Ser	Gln	Gly	Ile
61	CCT	CTC	TCT	AGG	AAC	ACA	CGC	TGC	ACC	TGC	ATC	GAG	ATC	AGT	AAT	GGA	TCT	GTT	AAT	CCA
21	Pro	Leu	Ser	Arg	Asn	Thr	Arg	Cys	Thr	Cys	Ile	Glu	Ile	Ser	Asn	Gly	Ser	Val	Asn	Pro
121	AGG	TCC	TTA	GAA	AAA	CTT	GAA	CTG	ATT	CCT	GCA	AGT	CAA	TCC	TGC	CCA	CGT	GTC	GAG	ATT
41	Arg	Ser	Leu	Glu	Lys	Leu	Glu	Leu	Ile	Pro	Ala	Ser	Gln	Ser	Cys	Pro	Arg	Val	Glu	Ile
181	ATT	GCC	ACA	ATG	AAA	AGG	AAT	GGG	GAG	AAA	AGA	TGT	CTG	AAT	CCA	GAA	TCT	AAG	ACC	ATC
61	Ile	Ala	Thr	MET	Lys	Arg	Asn	Gly	Glu	Lys	Arg	Cys	Leu	Asn	Pro	Glu	Ser	Lys	Thr	Ile
241	AAG	AAT	TTA	CTG	AAA	GCA	ATT	AAC	AAG	CAA	AGG	ACT	AAA	AGA	TCT	CCT	CGA	ACA	CAG	AAA
81	Lys	Asn	Leu	Leu	Lys	Ala	Ile	Asn	Lys	Gln	Arg	Thr	Lys	Arg	Ser	Pro	Arg	Thr	Gln	Lys
301	GAG	GCA	TAA																	
101	Glu	Ala	***																	

Fig. S2 Nucleotide and deduced amino acid sequences of cDNA encoding CXCL10 gene from Ujumqin sheep. The Cys which was showed in yellow represented 4 conservative cysteine residues in CXCL10 protein, the asterisk (***) represents stop codon

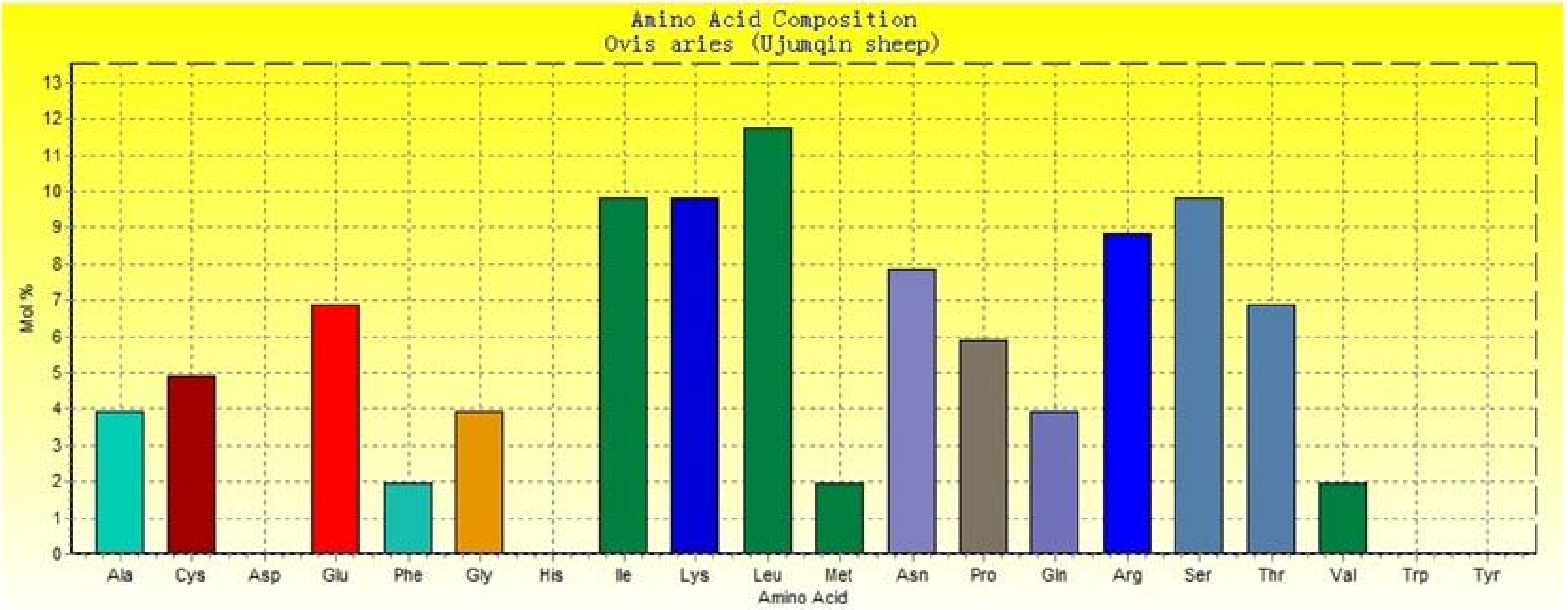

Fig. S3 Amino acid composition of CXCL10 gene from Ujumqin sheep

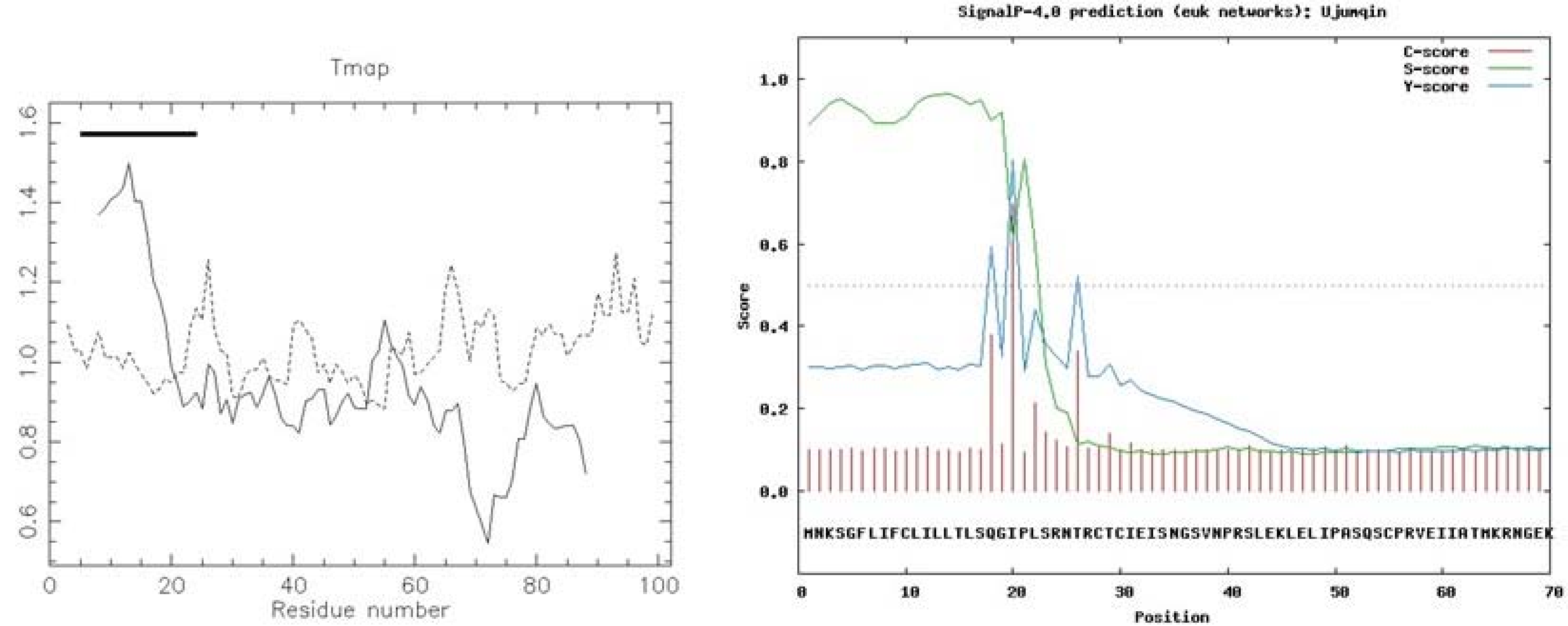

a b

Fig. S4 Prediction of transmembrane domain and signal peptide of CXCL10 protein of Ujumqin sheep. **a** the abscissa represents the number of residues, the ordinate represents hydrophobicity, high hydrophobicity occurred in the first 28 residues, indicating a probable transmembrane domain. **b** cleavage site of CXCL10 protein was between pos. 19 and 20, mean S-score in pos.1-19 was 0.928

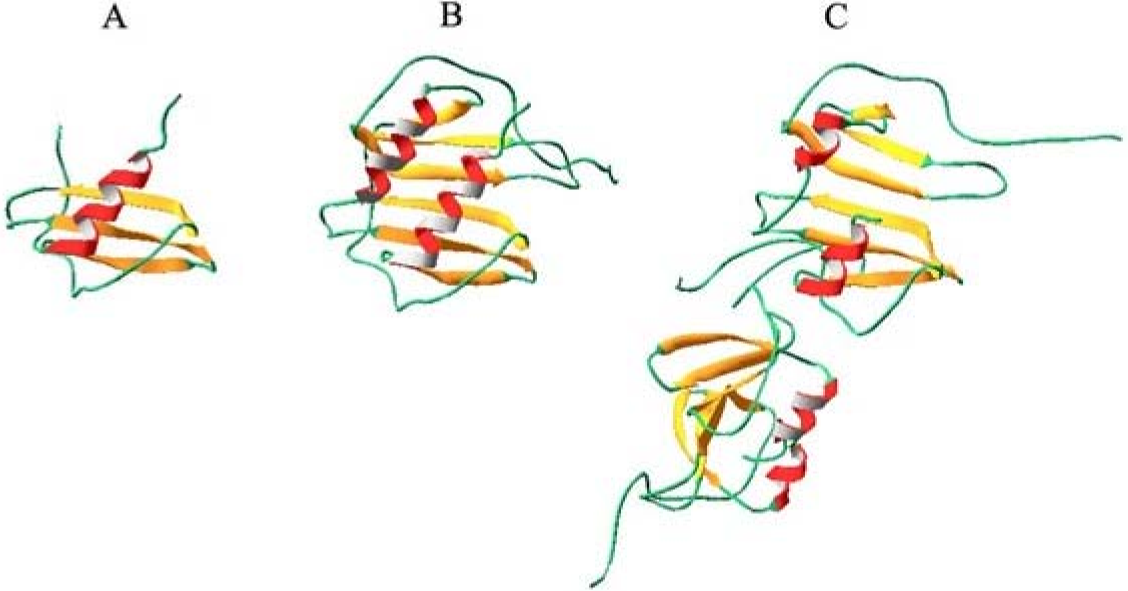

Fig. S5 CXCL10 protein spatial structure Magic Fit. **a** Ujumqin sheep, **b** Homo sapiens 1O80, **c** Mus musculus 2R3Z

MNKSGFLIFCLILLTLSQGIPLSRNTRCTCIEISNGSVNPRSLEKLELIPASQSCPRVEIIATMKRNGE
KRCLNPESKTIKNLLKAINKQRTKR SPR TQKEA

Fig. S6 Intra-domain features of CXCL10 amino acid sequences of Ujumqin sheep. ____: N-glycosylation site, : N-myristoylation site, : Casein kinase II phosphorylation site, : Protein kinase C phosphorylation site, : Small cytokines C-x-C subfamily signature
